# Reverse Engineering of Free-Form Surface Based on the Closed-Loop Theory

**DOI:** 10.1155/2015/903624

**Published:** 2015-03-24

**Authors:** Xue Ming He, Jun Fei He, Mei Ping Wu, Rong Zhang, Xiao Gang Ji

**Affiliations:** ^1^Department of Jiangsu Province Key Laboratory of Advanced Food Manufacturing Equipment and Technology, Jiangnan University, Wuxi 214122, China; ^2^School of Mechanical Engineering, Jiangnan University, Wuxi 214122, China; ^3^School of Science, Jiangnan University, Wuxi 214122, China

## Abstract

To seek better methods of measurement and more accurate model of reconstruction in the field of reverse engineering has been the focus of researchers. Based on this, a new method of adaptive measurement, real-time reconstruction, and online evaluation of free-form surface was presented in this paper. The coordinates and vectors of the prediction points are calculated according to a Bézier curve which is fitted by measured points. Final measured point cloud distribution is in agreement with the geometric characteristics of the free-form surfaces. Fitting the point cloud to a surface model by the nonuniform B-spline method, extracting some check points from the surface models based on grids and a feature on the surface, review the location of these check points on the surface with CMM and evaluate the model, and then update the surface model to meet the accuracy. Integrated measurement, reconstruction, and evaluation, with the closed-loop reverse process, established an accurate model. The results of example show that the measuring points are distributed over the surface according to curvature, and the reconstruction model can be completely expressed with micron level. Meanwhile, measurement, reconstruction and evaluation are integrated in forms of closed-loop reverse system.

## 1. Introduction

Free-form curves change freely in complex form, not like the law curve which can be described by the analytic functions. So, free-form surfaces also change in complex form; the reverse of objects with free-form surface is a research hot spot [1, 2]. The current reverse of products can be able to get the CAD model after data collection, data processing, and model reconstruction. These processes are sequentially performed from data acquisition to model reconstruction in the open-loop state, which makes data collection error be always present in the whole process of reverse engineering and be passed to the final CAD model [3, 4].

Huang and Qian [5, 6] proposed the reconstructed method of dynamic surface model, based on the point clouds of curve that have been achieved, using Kalman filtering method to guide B-spline surface model reconstruction dynamically. That is, the acquisition of point data is dynamically combined with B-spline surface reconstruction. This method can make the data point parameterization, dynamically determine next best measurement points and can effectively measure and reconstruct model with low differences. However, the whole process has a large amount of computation, data redundancy, and low efficiency and cannot evaluate the quality of the reconstructed model in time. Evaluating the quality of reconstruction and modification can only be carried on after the reconstruction model was manufactured, by comparing this production with primary product. If the reconstruction model can be directly evaluated, the accuracy can be ensured, the costs can be significantly reduced and the cycle of the reverse engineering is shortened as well.

Based on this, we propose a closed-loop reverse method which includes measurement, reconstruction, and evaluation, using OpenGL 3D graphics library to realize visualization in Visual C++ compiler environment. First, the contact CMM adaptive captures point cloud data based on curvature characteristics of the tested surface. Then fit point cloud to obtain surface model and calculate the error of check points on the fitting model and the corresponding points on the physical. If the error exceeds the threshold (the threshold is set according to the required accuracy), the actual measured value of the check points will be added to old point cloud, fitting model and checking again and updating fitted model until it meets the accuracy requirements. This method can reduce the error of data collected from the source and the error of reconstruction model and reverse free-form surfaces with high-precision and high efficiency.

## 2. Free-Form Surfaces Reverse Based on the Closed-Loop Theory

### 2.1. The Process of Reverse Designing Based on the Closed-Loop Theory

**Figure 1:**
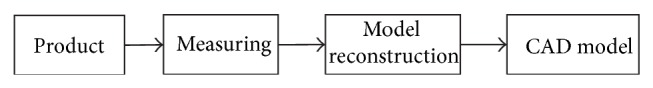
Traditional reverse process.

Conventional reverse process is an open-loop process as shown in Figure 1. Data collection and model reconstruction are independent of each other and are done in sequence.

This paper will bring the closed-loop theory into reverse process in order to contact data acquisition with CAD model reconstruction by online evaluation, which can make measurement and reconstruction with closed-loop. Model reconstruction is based on measured point cloud, meanwhile the model guides data supplement.

**Figure 2:**
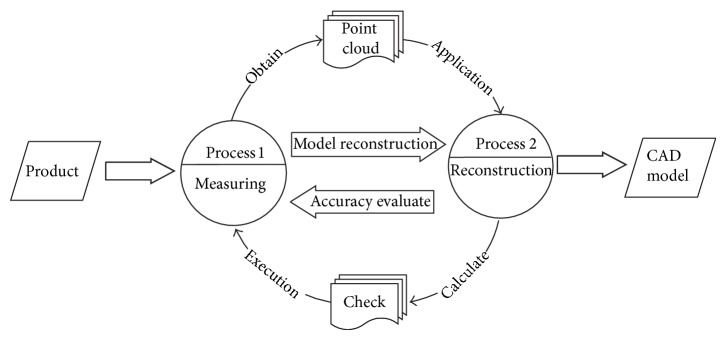
Reverse process based on the theory of closed-loop.

The concrete practice of the reverse engineering based on the theory of closed-loop is as following. Reconstruct surface model by using the initial measured point cloud and then extract some check points from the reconstructed surface model and measure actual value of those check points on the physical surface. Judge the accuracy of model whether to meet request by the contrast analysis of the check point error between the theoretical value and the actual value. If the error is less than the threshold, reconstructed model can be considered reliable and its accuracy can meet the requirements. Otherwise, the actual value of the check points must be added to the existing point cloud and fitted again, increase the density of new check points. Calculate theoretical value of those new check points and then measure those new check points and calculate its error of the theoretical value and the actual value; repeat this process until the model accuracy can meet the requirements. This method can improve model accuracy and avoid feature omissions. The principle of this method is shown in Figure 2.

This method can do online evaluation in the situations that the coordinate system for the measurement does not change. The evaluation results can visually reflect whether reconstructed surface is faithless and whether some feature data points are lost or away from the group. It can also reflect the effect of noise and can help extract unreliable model timely. The reverse process can be able to ensure the quality and accuracy, at same time its cycle will be shortened.

**Figure 3:**
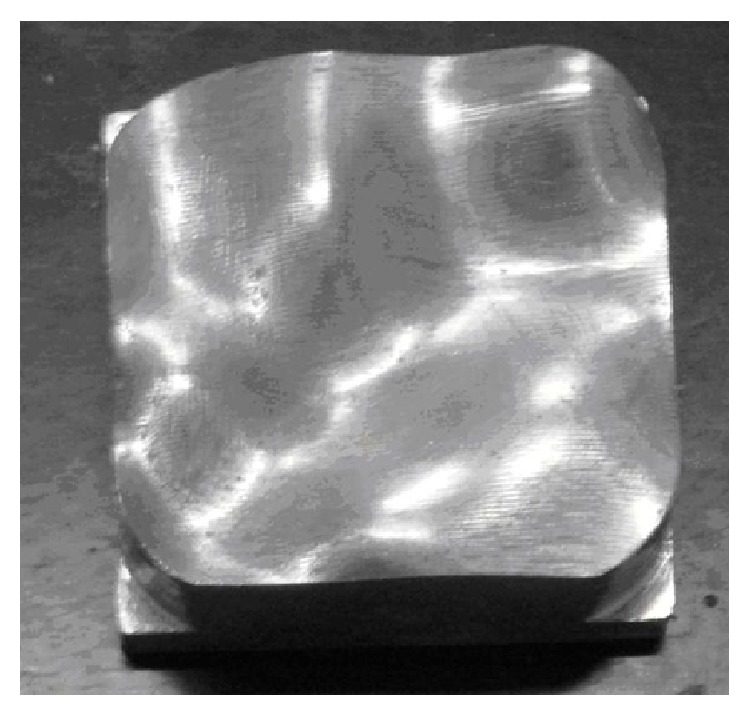
The product has free-form surface features.

Figure 3 shows a part with free-form surface characteristics and will be used in this method. It contains three steps: CMM adaptive measurement, surface model reconstruction, and online evaluation.

### 2.2. CMM Adaptive Measurement

The main types of data acquisition in the reverse process are contact and noncontact. Though noncontact measurement has high-efficiency, but it has lower accuracy. In this paper, touch-trigger CMM will be used to obtain point; the type of point data is scan lines style.

During the measurement, use the measured point fit a curve. Calculate the coordinates and vector direction of the predicted point according to the curvature characteristic of the curve. Then guide the CMM accurate measurement and obtain the actual value of the predicted point automatically.

**Figure 4:**
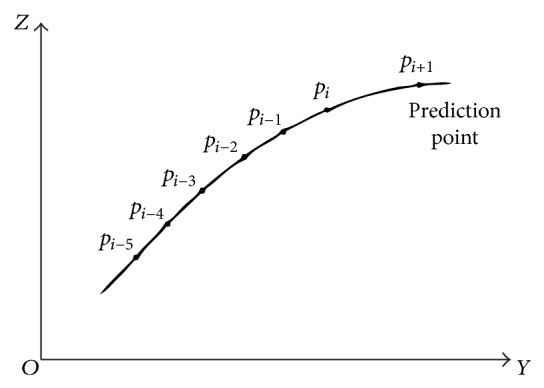
Adaptive measurement principle.

Quintic Bézier curve has second derivative at any point of the curve and sequential, suitable for fitting scanning lines point cloud on most product surface.  Figure 4 is the schematic of the Bézier curvature continuous adaptive measurement. Before adaptive measurement, some boundary points, the highest point, and the lowest point of the target surface should be measured by hand, the boundary points will construct four boundaries, and all adaptive measurement points must in the range of the boundaries. The coordinate of scan lines are calculated according to these boundaries. Assume initial measured points of every scan line are *p*_*i*−5_, *p*_*i*−4_, *p*_*i*−3_, *p*_*i*−2_, *p*_*i*−1_, *p*_*i*_, and these points are equally spaced, the distance of every two points and the vector direction of measurement of these six initial points are defined by operator, for example, *Z* direction. The six initial points *p*_*i*−5_, *p*_*i*−4_, *p*_*i*−3_, *p*_*i*−2_, *p*_*i*−1_, *p*_*i*_ would be fitted to form a quintic Bézier curve [7], as follows, *t* is Parameter and *t* ∈ [0,1]:

(1) Ct=∑i=055!5−i!i!1−t5−itipi=∑i=05B5,itpi.

This curve is assumed in plane *YOZ*, it can be decomposed in *Y*, *Z* directions as follows:

(2) $$\begin{array}{c} y=\alpha \left(t\right),\;z=\beta \left(t\right),\;t\in \left[0,1\right]. \end{array}$$

Curvature at any point on the curve:

(3) $$\begin{array}{c} k=\frac{\left|\alpha^{\prime }\left(t\right)\beta^{\prime \prime }\left(t\right)-\alpha^{\prime \prime }\left(t\right)\beta^{\prime }\left(t\right)\right|}{{\left[\alpha^{\prime 2}\left(t\right)+\beta^{\prime 2}\left(t\right)\right]}^{3/2}}. \end{array}$$

The radius of curvature is as follows:

(4) $$\begin{array}{c} \rho =\frac{1}{k},\;t\in \left[0,1\right]. \end{array}$$

The *p*_*i*+1_ is predicted point, and it is calculated by the fitted curve. From the nature of Bézier curve we know that first control point is the curve starting point and end control point is the curve end point. When *t* = 1, the curvature radius of the curve can be obtained at the curve end point. The larger the radius of curvature, the smaller the sampling step. The smaller the radius of curvature is, the larger the sampling step is.

**Figure 5:**
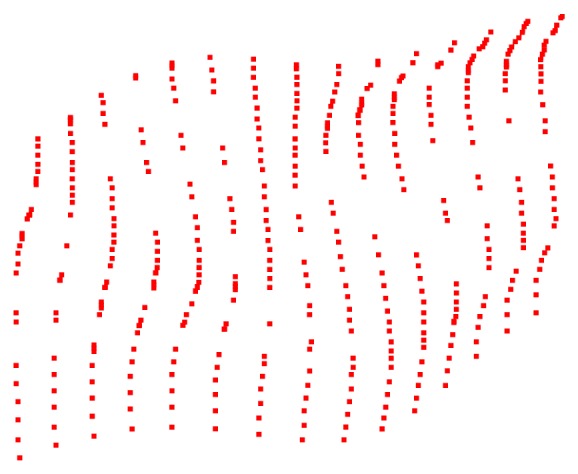
Point cloud by CMM adaptive measurement.

The Bézier curve is tangent to first and end of the edge of characteristic polygon. Assume the normal of point *p*_*i*_ is the vector direction of the predicted point *p*_*i*+1_. After actual value of point is measured by CMM, then delete point *p*_*i*−5_. The latest six points are *p*_*i*−4_, *p*_*i*−3_, *p*_*i*−2_, *p*_*i*−1_, *p*_*i*_, *p*_*i*+1_. Fit a new five Bézier curve and calculate point *p*_*i*+2_. Repeat this process until this scan line is measured completely and then start to measure other scan line. The measuring point cloud shows in Figure 5.

Distribution of the measured point cloud is based on surface characteristics. Measuring points distribute densely in changeful areas and are small and sparsely in gentle areas. The number of initial measurement points is 419. Thus, the most complete information can be expressed with minimal points [8].

### 2.3. Real-Time Surface Model Reconstruction

Low-level B-spline surfaces are closer to data points relative to the high prices without shock and warp and better reflect the actual characteristics of the real thing. Therefore, this study uses three B-spline curves and surfaces to fit point clouds directly and obtain surface model [9]. In order to meet the conditions of fitting nonuniform B-spline surface, this paper interpolates each scan line point cloud with some nonuniform B-spline curves and resample in each interpolation curve to obtain data points with evenly distribution and quantity consistently.

If data points (*m* + 1)×(*n* + 1) were topology rectangular array on the scanning lines *n* + 1, direction between the scanning lines and the scan lines of Figure 4 is assumed to be *u* direction and along the direction of the scan lines is *v* direction. The frequency of *u* and *v* direction is marked *k* and *l* and assumes that the number of *k* and *l* is three. It can define a 3 × 3 nonuniform B-spline surface as follows [10]:

(5) $$\begin{array}{c} p\left(u,v\right)=\overset{m}{\underset{i=0}{\sum }}\;\overset{n}{\underset{j=0}{\sum }}d_{i,j}N_{i,3}\left(u\right)N_{j,3}\left(v\right),\;u,v\in \left[0,1\right]. \end{array}$$

Use De Boer recursive formula [11] to calculate B-spline basis *N*_*i*,3_(*u*)  (*i* = 0,1,…, *m*) and *N*_*j*,3_(*v*)  (*j* = 0,1,…, *m*) according to knot vector *u* and *v*.

Construct nonuniform knot vector by using cumulative chord length method to guarantee the same type of distribution of knot vector and data points, that is, to ensure that the reconstruction of B-spline curves has higher quality. To ensure the characteristics of data points with interpolation, take clamped condition of quadruple knot endpoint. Mathematical expression of cumulative chord length parameterization is as follows:

(6) $$\begin{array}{c} u_{0}=u_{1}=u_{2}=u_{3}=0,\\ u_{j}=\frac{\sum_{i=1}^{j-3}L_{i}}{S},\;j=4,5,\ldots ,m+2,\\ u_{m+3}=u_{m+4}=u_{m+5}=u_{m+6}=1. \end{array}$$

**Figure 6:**
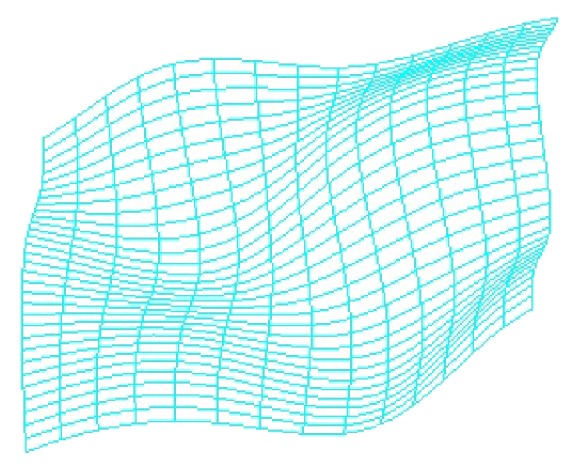
The surface's control grid.

*u*_*j*_ is a knot. *L*_*i*_ is distance between adjacent data points and *S* is the total length of the polyline posed by data points. Knot vector and topology rectangular array point cloud will decide the surface control point grid *d*_*i*,*j*_  (*i* = 0,1,…, *m*;  *j* = 0,1,…, *n*) by (5) [12] and the reconstruction surface's control grid is as shown in Figure 6.

### 2.4. Online Evaluation

In this paper, an evenly distributed method is proposed to determine the check points, dividing the curved surface model into the grid type, extracting the center of each grid as check point. The number of check points *H* is determined by the amount of scanning lines *L* and check times *r*, as the specific relationship of (7). When the check times increased once, the number of check points has about a fourfold increase. The check precision increases and the time becomes longer with the addition to the quantity of checks [13, 14], and check times up to three times can reach micron-level precision in experimental process.

(7) $$\begin{array}{c} H=\left(L\times 2^{r-1}\right)\left(L\times 2^{r-1}\right). \end{array}$$

Suppose that ${\overset{⌢}{p}}_{i}\;\;\left(i=0,1,\ldots ,H\right)$ represent check points extracted from reconstructed model, of which the coordinates and the normal vector information of curved surface where the points locate are used to instruct CMM to measure, getting the actual values of these points represented as *p*_*i*_  (*i* = 0,1,…, *H*), Subsequently, the error of reconstruction model is as follows:

(8) $$\begin{array}{c} e=\frac{\sum_{i=0}^{n}\left|p_{i}-{\overset{⌢}{p}}_{i}\right|}{H+1}. \end{array}$$

**Figure 7:**
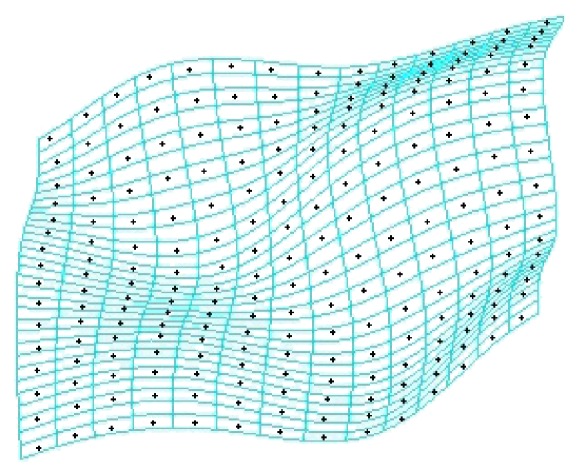
First check points.

Figure 7 shows the check points (black points) extracted from the curved surface model for the first time. In order to ensure the accuracy of inspection, check points cannot be same as the data points which are used for fitting.

Online accuracy evaluation of model is an important part of closed-loop reverse. If *e* is less than threshold (as in this example the threshold is 0.007 mm), it indicates that the reconstruction model is reliable and can be accepted. If *e* is greater than the threshold, the actual measured value of the check points in Figure 7 must be added to point cloud in Figure 5 to fit model again. Then, the curved surface grid is subdivided further according to (7) new check point for another inspection and evaluation is extracted.

**Figure 8:**
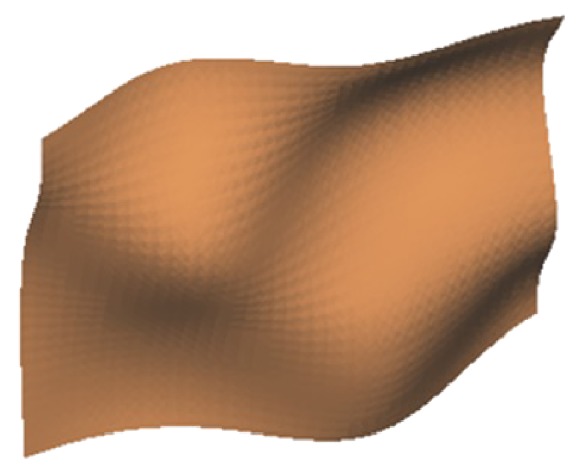
Final surface model.

**Figure 9:**
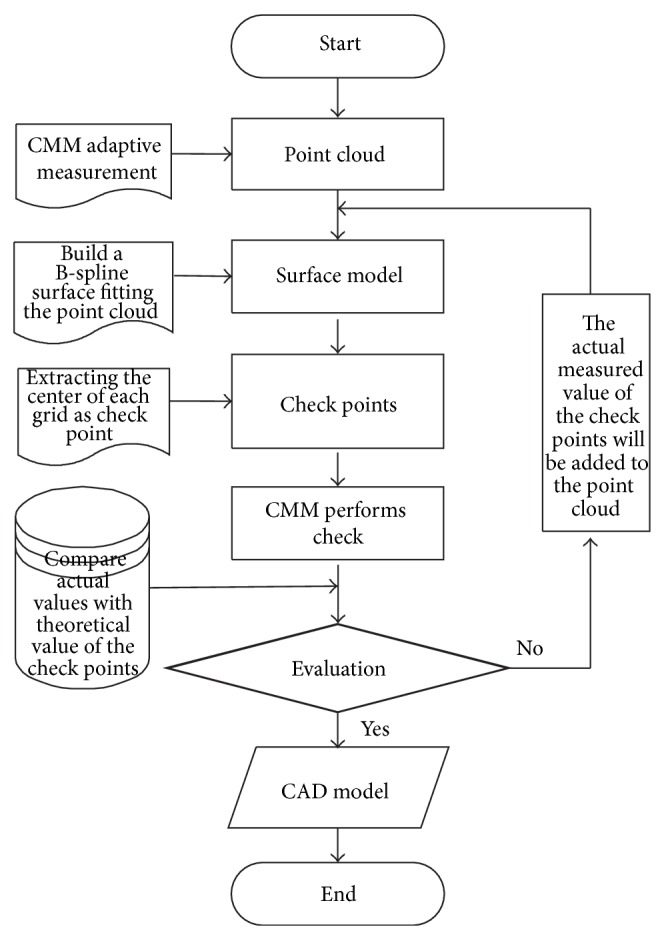
Flowchart of the closed-loop reverse engineering.

This process is repeated until *e* becomes less than the threshold, and the closed-loop reverse process is completed. In this case, the initial evaluation result of mouse surface is that *e*_1_ is greater than the threshold, and the number of first check points is 225. The second evaluation result is *e*_2_ = 0.0068 < 0.007, and the number of second check points is 900, and the overall operating time is 126 minutes, so this model is considered acceptable, as shown in Figure 8. The specific procedure of free-form curved surface measurement, reconstruction and evaluation of the closed-loop reverse system is shown in Figure 9.

## 3. Experimental Results

### 3.1. Free-Form Surface Reconstruction Algorithm Verification

Because the mathematical model of saddle surface is known, it is chosen as test object in order to inspect the reliability of the free-form surface reconstruction algorithm. The mathematical equations for the saddle surface are

(9) z=x−15216−y−21.8301225+5  x∈0,30,  y∈0,43.6602.

**Figure 10:**
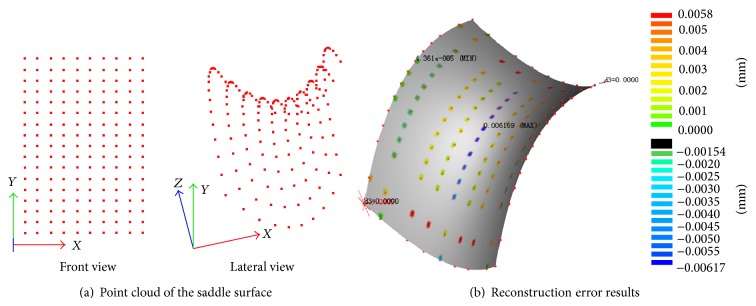
Reconstruction algorithm verification.

Figure 10(a) shows that a group of original points are calculated by mathematical saddle surface equation to reconstruct surface. First, the original points are fitted into a surface with B-spline curve and the surface reconstruction algorithm. Then, select a group of check points on this surface model. Finally, compare the check points with the corresponding points calculated by mathematical equation and get the error. The comparison results are shown in Figure 10(b). The maximum error is 0.00617 mm, which is micron-level. We can arrive at a conclusion from the results that this reconstruction algorithm is applicable to reverse modeling free-form surface products.

### 3.2. Examples of Applications

**Figure 11:**
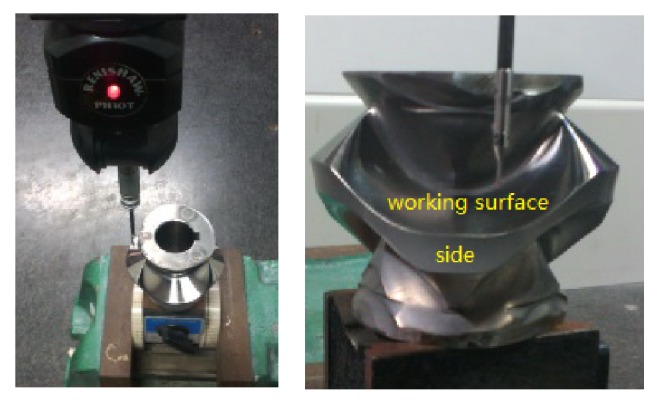
The arc surface cam.

Figure 11 shows a precise arc surface cam. Its face area is G1 continuous, and side area is G2 continuous, while the intersection is G0 continuous. All of these form a complex composite surface. The working surface should be divided into some patches according to the principle that the curvature is continuous and then measured by the adaptive measurement method with CMM. The side can be measured adaptively due to curvature being continuous.

**Figure 12:**
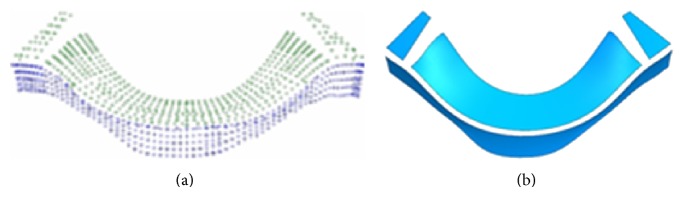
Point cloud and patches. (a) Measured points by CMM. (b) Patches by fitting the point cloud.

**Figure 13:**
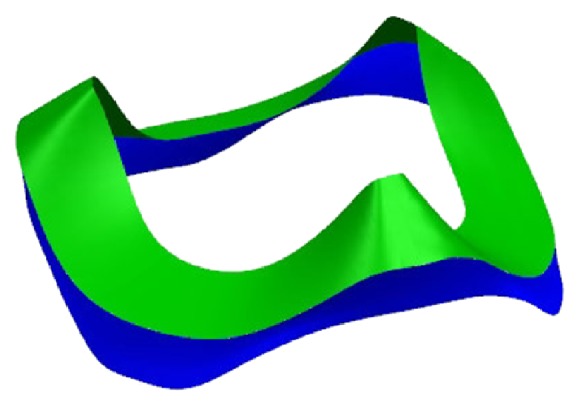
CAD model.

Figure 12 shows the point cloud data result from measuring the arc surface cam, and four patches are fitted with these point cloud data (for the convenience of reading, Figure 12 shows only about 1/3 of the point cloud and patches). The number of measurement points is 2655 and the operating time is 216 minutes. After fitting, the patches are evaluated for the first time, the number of first check points is 1532 and operating time is 125 minutes, the maximum *e* of working surface is 0.1070 mm, and the maximum *e* of side is 0.2944 mm. As a result, the first reconstruction patches are acceptable. Blend the six top patches to make a working surface, and the side patches to make a side surface and then extend and trim the working surface and side surface to obtain an entire model. Because the boundary of the cam cannot be measured by CMM, the quality and accuracy of blend faces cannot be controlled; just revise the blend faces according to the patches and ensure the entire surface's smoothness. The final CAD model is shown in Figure 13.

## 4. Conclusions

This paper has proposed a new reverse method based on the theory of closed-loop, achieving the information interaction between the measurement, reconstruction and evaluation. It avoids the separation between actual measurement, reconstruction and evaluation in traditional reverse process, and comparative evaluation can be carried out without reconstructed model manufactured. Aimed at the objects with free-form surface, that the geometric characteristics of real shape guide CMM to measure adaptively can come true, which results in the point cloud data with distribution reasonable and appropriate number. The surface model is obtained by using the method of non-uniform B-spline, on-line evaluated and updating model instead of evaluation after manufacturing. Finishing the process, precise measurement point cloud and accurate CAD model is obtained ultimately. The experimental results indicate that measurement accuracy and reconstruction accuracy of this method can reach micron-level. Additionally, the cost is evidently reduced, and the cycle is shortened obviously as well. Moreover, there is no need to make reconstruction model to compare and evaluate.
